# Parallels in Processing Boundary Cues in Speech and Action

**DOI:** 10.3389/fpsyg.2019.01566

**Published:** 2019-07-16

**Authors:** Matt Hilton, Romy Räling, Isabell Wartenburger, Birgit Elsner

**Affiliations:** ^1^Department of Psychology, Cognitive Sciences, University of Potsdam, Potsdam, Germany; ^2^Department of Linguistics, Cognitive Sciences, University of Potsdam, Potsdam, Germany

**Keywords:** Closure Positive Shift (CPS), Event-related Potentials (ERP), speech segmentation, action segmentation, prosodic boundary cues, prosody processing, kinematic boundary cues, action processing

## Abstract

Speech and action sequences are continuous streams of information that can be segmented into sub-units. In both domains, this segmentation can be facilitated by perceptual cues contained within the information stream. In speech, prosodic cues (e.g., a pause, pre-boundary lengthening, and pitch rise) mark boundaries between words and phrases, while boundaries between actions of an action sequence can be marked by kinematic cues (e.g., a pause, pre-boundary deceleration). The processing of prosodic boundary cues evokes an Event-related Potentials (ERP) component known as the Closure Positive Shift (CPS), and it is possible that the CPS reflects domain-general cognitive processes involved in segmentation, given that the CPS is also evoked by boundaries between subunits of non-speech auditory stimuli. This study further probed the domain-generality of the CPS and its underlying processes by investigating electrophysiological correlates of the processing of boundary cues in sequences of spoken verbs (auditory stimuli; Experiment 1; *N* = 23 adults) and actions (visual stimuli; Experiment 2; *N* = 23 adults). The EEG data from both experiments revealed a CPS-like broadly distributed positivity during the 250 ms prior to the onset of the post-boundary word or action, indicating similar electrophysiological correlates of boundary processing across domains, suggesting that the cognitive processes underlying speech and action segmentation might also be shared.

## Introduction

While still relatively rare, interdisciplinary examination of speech and action processing is vital given the striking parallels between the two domains: Both speech and action consist of sub-units that are sequentially and hierarchically organized, meaning that speech and action productions inflate over time and can be –in principle endlessly– concatenated. The listener or observer must process this continuous stream of information, encode it, and segment it into meaningful sub-units before being able to interpret it. In both domains, bottom-up processes (analyzing perceptual cues) as well as top-down (contextual) processes support the segmentation of an utterance or an action sequence, allowing for the extraction of their underlying structure and meaning (Zacks, 2004; Goyet et al., 2016; Emberson, 2017). These parallels in the structure of and processes operating on the information streams suggest that the segmentation of speech and action might rely on domain-general cognitive processes.

Much work has examined the bottom-up processes that guide segmentation of the speech stream into lexical or syntactic sub-units, with a focus on the acoustic cues that mark boundaries between these sub-units. Specifically, three main prosodic boundary cues have been found to phonetically mark the edges of major prosodic boundaries in German and across different languages (for review, see Wagner and Watson, 2010; Cole, 2015; for German, see, e.g., Kohler, 1983; Peters, 2005; Kentner and Féry, 2013; Petrone et al., 2017). The most salient durational prosodic boundary cue is a *pause*, an interval of silence. This pause is often accompanied by a *lengthening* and by a change in the fundamental frequency f0 (*pitch rise*) of the immediate pre-boundary segments. Boundaries in German speech are most often marked by a combination of these prosodic boundary cues, but individual cues alone or a combination of two cues have also been found to mark a prosodic boundary (Peters, 2005). Major prosodic boundaries (so-called *intonation phrase boundaries*) often coincide with boundaries of syntactic clauses (Downing, 1970; Selkirk, 1984; Nespor and Vogel, 1986; for German, see Truckenbrodt, 2005). Infants track these bottom-up prosodic boundary cues, with the close prosody-syntax mapping supporting the infant’s developing understanding of syntactic structures (so-called *prosodic bootstrapping*, e.g., Gleitman and Wanner, 1982; Nazzi et al., 2000; for review, see Speer and Ito, 2009). Furthermore, adult listeners make use of prosodic cues when syntactic and lexical structures do not provide sufficient information to guide segmentation (e.g., Marslen-Wilson et al., 1992; Warren et al., 1995; Schafer et al., 2000).

A well-known electrophysiological correlate of speech segmentation is the Event-related Potential (ERP) component *Closure Positive Shift* (CPS; Steinhauer et al., 1999), which is related to the processing of a prosodic boundary by adult native speakers across several languages (Pannekamp et al., 2005; Holzgrefe et al., 2013; Holzgrefe-Lang et al., 2016; for review, see Bögels et al., 2011a). The CPS constitutes a slow, broadly distributed positivity over central and parietal electrodes that starts around the onset of a prosodic boundary and lasts approximately 500 ms, or until the onset of the subsequent word (Bögels et al., 2011b). Importantly, the CPS has been found (albeit with a slightly differing scalp distribution) at the closure of a prosodic phrase also in auditory jabberwocky or pseudo-word sentences, as well as for f0 changes and pauses in auditory stimulus material without syntactic or lexical information (hummed speech; Pannekamp et al., 2005), indicating that the CPS likely reflects the bottom-up processing of perceptual information. Glushko et al. (2016) found a language-like CPS at the onset of phrase boundaries in music, in both musicians and non-musicians (but see Knösche et al., 2005), and therefore expanded the definition of the CPS as reflecting the processing of a “closure of a grouped perceptual structure” (Glushko et al., 2016, p. 23). Furthermore, the CPS is not restricted to auditory material, since it occurs at the closure of a prosodic phrase when participants silently read visually presented sentences (Steinhauer, 2003; Hwang and Steinhauer, 2011; but see Kerkhofs et al., 2008, for an alternative account), and Gilbert et al. (2015) accordingly argued for a more domain-general understanding of *Positive Shifts*, associating them with domain-general perceptual chunking linked to short-term memory.

A close reading of past research indicates many parallels between the processing of boundaries in speech and action. For example, like speech, action sequences are reliably segmented into sub-units (individual actions), as confirmed by high inter-rater agreement on the location of the boundaries between these sub-units in everyday action sequences (e.g., clearing a cluttered table; Newtson et al., 1977). Furthermore, the boundaries between sub-units of an action sequence are highly salient, as demonstrated by the findings that adults attend preferentially to movements occurring between rather than within sub-units of an action sequence (Hard et al., 2011), and that memory for the individual actions that form the action sequence is disrupted when boundaries between them are removed (Schwan and Garsoffky, 2004). Thus, just as prosodic boundary cues support the correct parsing of spoken language, boundaries within action sequences seem to play an important role in action sequence perception.

In a further parallel with prosodic boundary processing, perception of the boundaries in action sequences also relies in part on low-level perceptual cues. Specifically, recent work has shown that kinematic properties of the actions that form the sequence can signal the location of a boundary between sub-units. For example, a change in motion velocity occurs at the boundary between sub-units of action sequences, (rapid acceleration/deceleration; Zacks et al., 2009; McAleer et al., 2014) suggesting that changes in speed of the movement around the time of the boundary offer a kinematic cue to the location of the boundary within the sequence. A pause in an action sequence (i.e., a motionless interval) can also be a kinematic cue that signals a boundary between actions: Participants report the use of pauses to determine boundaries between actions (Bläsing, 2014) and expect pauses to occur at boundaries in action sequences (Friend and Pace, 2016). The similarity of the low-level cues (i.e., change in duration of the pre-boundary unit, presence of a pause) raises the prospect that the cognitive processes involved in the perception of these kinematic boundary cues are similar to those involved in prosodic boundary cue processing.

Notably, sign languages also use kinematic cues to express prosodic functions, for instance a reduced velocity and phrase-final lengthening (see e.g., Malaia et al., 2013). Sign languages have a complex hierarchical structure comprising the same linguistic levels as found in spoken languages, such as phonology, syntax, etc. (for a recent review see Goldin-Meadow and Brentari, 2017). It has, for example, been shown that across several sign languages, event telicity (telic verbs entail an endpoint, such as “to close”, while atelic verbs do not require endpoints, such as “to think”) is marked by the presence or absence of gesture boundaries, which are detected and interpreted even by non-signers (Strickland et al., 2015). For a recent discussion of a framework on joint neural and cognitive mechanisms in language, sign language, and action see Blumenthal-Dramé and Malaia (2019).

Another argument for the similarity of the involved processes comes from research showing that modulation of kinematic or prosodic boundary cues has similar effects on processing of the information stream as a whole. For example, exaggeration of the prosodic boundary cues can bolster speech perception (e.g., better segmentation of strings of pseudowords spoken with infant-directed vs. adult-directed pitch contours; Thiessen et al., 2005), and likewise, exaggerated kinematic boundary cues (e.g., extending the pause) can improve memory for the individual actions constituting a sequence (Gold et al., 2017). Furthermore, “pure prosody” can signal a boundary, as evidenced by the findings of prosodic boundary processing in nonsense speech or even hummed speech (Pannekamp et al., 2005), and kinematic boundary cues seem to operate similarly, because acceleration and speed changes are interpreted as a marker of a boundary when observed movements are not discernible as actions (e.g., when hand movements are displayed as an inverted moving constellation of point-lights; Hemeren and Thill, 2010). Thus, the perceptual cues that mark boundaries in speech and in action sequences seem robust enough to signal the presence of a boundary in a perceived sequence independent of any contextual information.

The aim of the current study was to further probe the similarity of processes underlying speech and action segmentation by examining the electrophysiological correlates of kinematic and prosodic boundary cue processing. Specifically, we examined whether the processing of kinematic boundary cues would evoke a positivity in the ERP, and whether this positivity would share temporal and spatial characteristics with the CPS that could be expected for the prosodic boundary cues. To do this, we recorded adults’ EEG while listening to spoken sequences of three verbs (co-ordinated with an “and”; auditory stimuli; Experiment 1) and while observing performed sequences of three actions (visual stimuli; Experiment 2). In each domain, participants were presented with sequences that did or did not contain perceptual cues marking a boundary following the critical second verb or action. Finding similar ERP components in response to a boundary in both domains, namely a broadly distributed positivity elicited by processing of the boundary, could be interpreted as the electrophysiological correlate of a domain-general response to the perceptual cues that mark the boundaries in continuous streams of speech and action.

## Materials and Methods

### Experiment 1: Processing of Prosodic Boundary Cues in Auditory Speech Sequences

#### Participants

For both experiments, participants were recruited from the student population of the University of Potsdam, Germany. Each participant was right-handed as confirmed by the Edinburgh Handedness Inventory (a German Version based on Oldfield, 1971) and had normal or corrected-to-normal vision. No participant reported a history of neurological, psychiatric, or hearing disorders. Participants gave informed consent and received either reimbursement or course credit for their participation.

The final sample for Experiment 1 consisted of 23 participants (11 females) with a mean age of 26.01 years (*SD =* 6.41 years; range: 19–44 years). Five additional adults were tested, but their data had to be excluded from analyses due to technical problems (*n* = 4) or failure to follow instructions (*n* = 1). None of the participants had taken part in Experiment 2.

#### Stimuli

**FIGURE 1:**
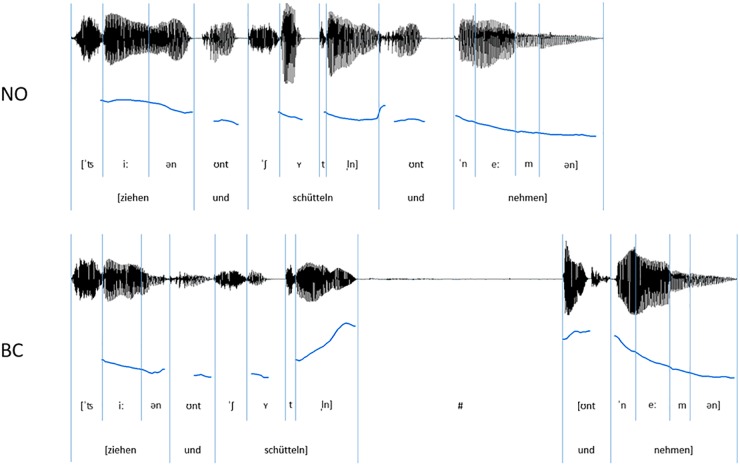
Exemplar oscillograms (black) and pitch contours (blue) of auditory speech stimuli (sequences of three spoken German verbs) for the NO-condition (without prosodic boundary) and the BC-condition (with a prosodic boundary signaling a boundary after the critical verb).

The auditory speech stimuli consisted of sequences of three disyllabic, trochaic German verbs co-ordinated by the conjunction *und* (and) (see for comparable stimuli with proper names or adjectives, e.g., Aasland and Baum, 2003; Holzgrefe et al., 2013). We used the following five verbs: *drehen* (to spin), *rollen* (to roll), *schütteln* (to shake), *ziehen* (to pull), and *nehmen* (to take) (see Figure 1). The stimuli were spoken by a young female German native speaker who had received professional vocal training, meaning that no specific German accent could be identified when listening to the stimuli. Stimuli were recorded in a soundproof booth with an audio-technical studio microphone (type AT4022a), using the open-source program Audacity (Version 2.1) and an M-AUDIO-Audiophile 2496 sound card at a sampling rate of 44100 Hz with 16-bit resolution. The speaker was instructed to convey the stimuli clearly and in an infant-directed manner. Two conditions were recorded: sequences of the NO-condition contained no prosodic boundary, while the sequences of the BC-condition contained a prosodic boundary starting at the second syllable of the critical second verb. The speaker was provided with a written list of the verb sequences in which the conditions were indicated by brackets and the position of the boundary by a hash (e.g., “[schütteln und drehen und rollen]” or “[schütteln und drehen] # [und rollen]”). As a sanity check, three naïve listeners confirmed the prosodic chunking of the stimuli, by reproducing the bracketing on a plain (i.e., without bracketing and a hash) list of verb sequences (accuracy was 100%).

The final stimulus set consisted of 120 verb sequences (see Figure 1 for example). Each of these unique sequences was present once without a prosodic boundary (NO-condition, 60 sequences) and once with a prosodic boundary (BC-condition, 60 sequences). The sequences had an average length of 1.75 s in the NO-condition and 2.79 s in the BC-condition. Within a condition, there was no repetition of single sequences^1^. Acoustic analyses were run using the open-source software for phonetic analyses PRAAT (Version 6.0.17; Boersma and Weenink, 2016). The audio files were scaled to a mean intensity of 70 dB. It was confirmed that the two conditions differed with regard to the acoustic parameters during and after the second verb (i.e., the critical verb, which did or did not contain boundary cues). The boundary in the BC-condition was characterized by three prosodic boundary cues: (1) a lengthening of the pre-boundary final syllable of the critical verb; (2) a pitch rise (f0-rise) of the pre-boundary syllable of the critical verb (difference of the measured maximal f0 of the last syllable sonorant and the minimal f0 on the first vowel of the second syllable); and (3) a silent pause after the critical verb and before the onset of the post-boundary word “und”. The values presented in Table 1 confirm the presence of all of the three expected prosodic boundary cues in the verb sequences of the BC-condition containing a prosodic boundary in comparison to the sequences of the NO-condition without a prosodic boundary. Example stimuli can be found at the Open Science Framework project page of this paper (Hilton et al., 2019).

**Table 1:** Mean values (*SD*; range) of the acoustic correlates of the prosodic boundary cues (measured during or after the second syllable of the critical verb) in the 120 recordings of natural speech used in the NO-condition (60 verb sequences without a prosodic boundary) and the BC-condition (60 verb sequences containing a boundary between the critical verb and the post-boundary word).

Prosodic boundary cue	Acoustic correlate	NO-condition	BC-condition
Pitch change (final syllable of the critical verb)	Pitch rise (Hz)	38 (28; 0–94)	259 (25; 208–317)
	Maximum f0 (Hz)	261 (16; 229–311)	412 (23; 369–469)
Lengthening (final syllable of the critical verb)	Final syllable duration (ms)	162 (38; 91–223)	229 (37; 164–301)
Pause (after the critical verb, before the onset of the post-boundary word “und”)	Pause duration (ms)	0	1026 (162; 593–1455)


#### Procedure

The experiment was run in a sound-attenuating chamber while the continuous EEG was recorded. The stimuli were presented auditorily using Presentation^®^ software (Version 19.0; Neurobehavioral Systems^2^). The participants were instructed to avoid eye blinks and body movements during stimulus presentation. To ensure that participants understood the instructions, the experimental session started with one practice trial from each condition. The maximum duration of the experiment was about 35 min.

While sitting in a comfortable chair, the participants listened to 240 auditory stimuli (60 sequences each in the NO- and the BC-condition, and 120 sequences with an unnatural boundary; see Footnote 1) presented via in-ear-headphones (E-A-RTONE 3A Insert Earphones, Aearo Technologies Auditory Systems, Indianapolis, IN). The order of the stimuli was pseudorandomized with the constraints that not more than two items of the same condition followed each other and the same combination of verbs never occurred across consecutive trials. To reduce eye movements, each trial started with a central fixation cross on the monitor lasting for 500 ms, after which the stimulus began playing. The central fixation cross remained on the monitor during stimulus presentation and for a further 700 ms following the offset of the final verb. Then, the two bracketed sequences ([X and Y] [and Z] vs. [X and Y and Z]) were displayed, and participants were required to indicate whether a boundary had or had not been present in the previous stimulus, via a button press on a response box (Cedrus RB-830 Response Pad^3^). Presentation side of the bracketed boundary and no-boundary sequences was counterbalanced across participants. Participants were required to respond within 3000 ms. The subsequent trial started after an inter-trial interval of 2500 ms. Participants took a short break halfway through stimulus presentation.

### Experiment 2: Processing of Kinematic Boundary Cues in Visual Action Sequences

#### Participants

Participants were drawn from the same pool as in Experiment 1, according to the same participant requirements. The final sample consisted of 23 participants (11 females) with a mean age of 26.17 years (*SD* = 7.60 years; range: 19–52 years). Five additional adults were tested, but their data had to be excluded from analyses due to technical problems (*n* = 3) or random responding in both conditions (*n* = 2). None of the participants had taken part in Experiment 1.

#### Stimuli

**FIGURE 2:**
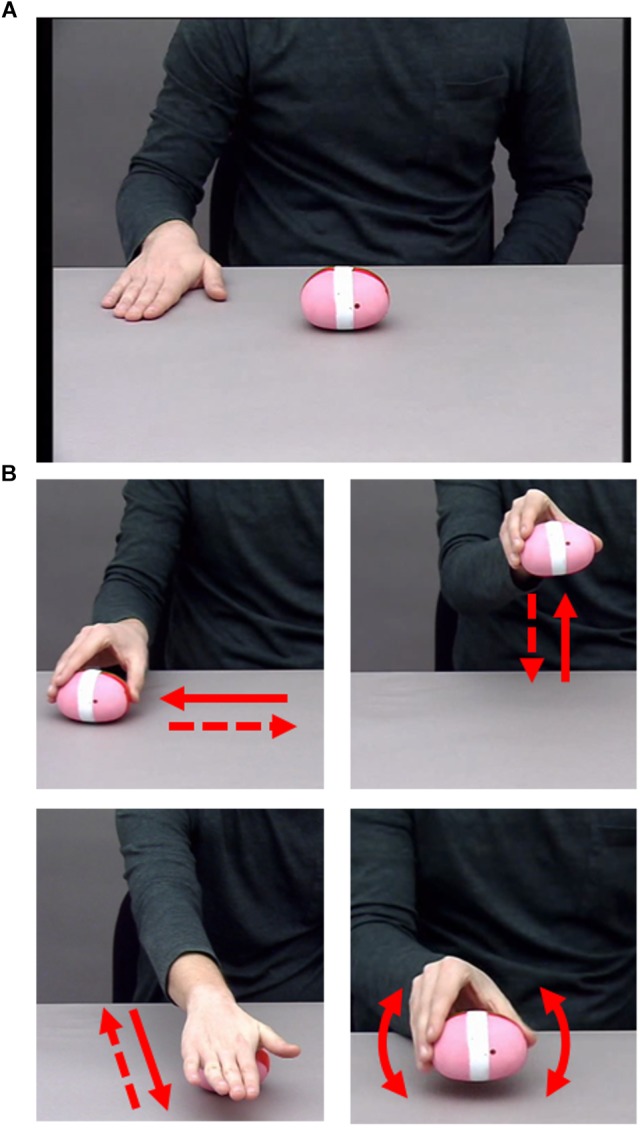
**(A)** Screenshot of starting position of each video from Experiment 2. **(B)** Close-up of each individual action, with arrows indicating the movement that formed each action. Clockwise from top left: sliding, lifting, shaking, rolling. Written informed consent was obtained from the actor allowing for publication of these images.

The visual action stimuli consisted of videos of an actor performing sequences of three hand actions on a balloon sand weight. We used the following four individual actions: lifting, rolling, shaking, and sliding (see Figure 2). All actions shared critical properties: They can be performed on the same object, they take place in the same approximate space, they cannot be performed simultaneously, and they can be performed in any order. Stimuli consisted of videos of an actor sat centrally at a table on which the object was placed. Only the actor’s right arm and hand, shoulders, and torso were visible (see Figure 2). Two conditions were recorded: Sequences of the NO-condition contained no kinematic boundary, while sequences of the BC-condition contained a boundary following the second action (i.e., the critical action, which either contained kinematic boundary cues or not). The actor was provided with a written list of the action sequences to be performed, and the presence of a boundary was indicated by the bracketing structure identical to that of Experiment 1. The actor was instructed to perform the actions in such a way that a naïve observer would be able to determine which condition each sequence belonged to. Each video began with a 1000 ms still frame in which the actor’s hand was placed to the side of the object, immediately followed by the movement of the hand toward the object to begin the 3-action sequence. Videos were recorded at 25 frames per second and at a resolution of 720 × 576 pixels. The sequences had an average length of 4.49 s in the NO-condition and 5.63 s in the BC-condition. Example stimuli can be found at the Open Science Framework project page of this paper (Hilton et al., 2019).

The final stimulus set consisted of 48 action sequences. Each sequence was presented once without a boundary (NO-condition, 4! = 24 sequences) and once with a boundary (BC-condition, 24 sequences). Within each set, each individual action appeared in each position. Frame-by-frame coding allowed an analysis of the movement of the object throughout each trial. The beginning of each action was defined as the time of the first frame in which the object was moved relative to the previous frame. The offset of each action was defined as the time of the first frame in which the object did not move relative to the previous frame (see Table 2). Unlike the auditory stimuli presented in Experiment 1, the action sequences often contained a short pause between sub-units even when no boundary was present, because the hand sometimes had to change grip in preparation for the next movement.

**Table 2:** Mean duration (*SD*; range) in milliseconds of the sub-units forming the 48 action sequences used in the NO-condition (24 action sequences without kinematic boundary) and the BC-condition (24 action sequences containing a kinematic boundary at/after the critical second action and before the final action).

	Action 1 (initial action)	Pause 1	Action 2 (critical action)	Pause 2	Action 3 (final action)
NO-condition	1265 (120; 1000–1600)	265 (236; 0–680)	1265 (212; 880–1520)	325 (346; 0–680)	1367 (172; 920–1760)
BC-condition	1298 (178; 1120–1680)	277 (230; 0–720)	1510 (185; 1240–2040)	1105 (239; 480–1840)	1437 (218; 1200–1880)


A repeated measures analysis of variance (ANOVA) on the duration of the single actions with the within-subjects factors action position (initial vs. critical vs. final) and condition (BC vs. NO) revealed significant main effects of action position, *F*(2, 46) = 6.74, *p* = 0.0027, $\eta_{G}^{2}$ = 0.82, and condition, *F*(1, 23) = 15.21, *p*< 0.001, $\eta_{G}^{2}$ = 0.095. Critically, a significant interaction, *F*(2,46) = 3.91, *p* = 0.027, $\eta_{G}^{2}$ = 0.062, was also revealed. Follow-up Bonferroni-corrected contrasts showed that only the duration of the critical action was significantly longer in the BC- than NO-condition, *p*< 0.001, but not the duration of either the initial action, *p* = 0.99, or final action, *p* = 0.61. Hence, the boundary in the BC-condition was signaled kinematically by pre-boundary lengthening. Similarly, a repeated measures ANOVA on the duration of the pauses with the within-subjects factors pause position (first, i.e., between the initial and critical action, vs. second, i.e., between the critical and final action) and condition (BC vs. NO) also revealed significant main effects of pause position, *F*(1, 23) = 35.73, *p*< 0.001, $\eta_{G}^{2}$ = 0.42, and condition, *F*(1, 23) = 107.47, *p*< 0.001, $\eta_{G}^{2}$ = 0.36 and a significant interaction, *F*(1,23) = 126.19, *p*< 0.001, $\eta_{G}^{2}$ = 0.35. Follow-up Bonferroni-corrected contrasts revealed that only the second pause (at the position of the boundary in the BC-condition) was significantly longer in the BC- than the NO-condition, *p*< 0.001, but not the duration of the first pause, *p* = 0.99. Therefore, although a pause was present between the critical and final action in both conditions, the longer pause was a further kinematic cue to signal the presence of a boundary in the BC condition.

#### Procedure

The procedure was identical to Experiment 1, except that instead of listening to auditory speech stimuli, participants viewed the visual action stimuli on a computer monitor. Participants were presented with four blocks of 48 trials, with each block comprising all videos in a random order, meaning that each participant saw 192 action sequences. Participants took a short break between blocks.

### EEG Recording, EEG Data Preprocessing, and ERP Data Analysis

EEG recording and data analysis were identical for both experiments. Continuous EEG was recorded while the participants listened to the auditory speech stimuli or watched the visual action stimuli, from 32 active Ag/AgCl electrodes (actiCAP, Brain Products, Germany) with a sampling rate of 1000 Hz. The electrodes were mounted in an elastic EEG cap according to the international 10–10 system (Epstein et al., 2006). The electrode Fp1 served as the ground electrode, and the left mastoid served as online reference and was re-referenced offline to averaged left and right mastoids. Eye blinks were detected by recording an electro-oculogram (EOG) with one electrode placed below, and one above the right eye (Fp2). Impedances of the electrodes were kept below 5 kΩ.

The EEG signal was preprocessed in BrainVision Analyzer (Version 2.1; Brain Products, Gilching, Germany). In order to remove slow drifts and muscle artifacts, we applied a digital bandpass filter ranging from 0.2 to 70 Hz and a notch filter at 50 Hz. We analyzed epochs of 2000 ms time-locked to the onset of the post-boundary word “und” or to the onset of the post-boundary action (-1500 to 500 ms) to distinguish between onset components of the post-boundary stimulus material and the ERPs elicited by the processing of the boundary (Glushko et al., 2016, for a similar procedure).

In each of the epochs, the EEG data were adjusted to a baseline 200 ms from the onset of the critical, pre-boundary verb^4^ or action. Eye blinks and movements were automatically detected by running an ocular correction based on the algorithm by Gratton et al. (1983). Further artifacts were inspected automatically (criteria: maximally allowed voltage step of 50 μV/ms, maximally allowed difference of values in intervals of 200 μV, lowest allowed activity in intervals of 0.5 μV). Epochs including artifacts were excluded from further analyses.

In line with previous research (e.g., Holzgrefe et al., 2013; Holzgrefe-Lang et al., 2016), EEG data were analyzed at three regions of interest: frontal (F3, Fz, F4), central (C3, Cz, C4), and posterior (P3, Pz, P4). Isolating and identifying the CPS from the ERP has previously posed difficulty, because the onset of the post-boundary word also typically evokes a positivity (P2), meaning that previous studies may have incorrectly assumed that this post-boundary onset component was evidence of a CPS (for a discussion see Männel and Friederici, 2009; Glushko et al., 2016). In order to avoid this difficulty, we analyzed mean ERP amplitude during the 250 ms interval prior to the onset of the post-boundary word “und” or final action, meaning that we can be certain that any effects on the ERP were related to the processing of the prosodic or kinematic boundary, and not the final sub-unit. This 250 ms interval was chosen to roughly match the duration of the short pause that followed the critical action in the NO-condition of Experiment 2. To allow for comparisons between the present results and previous findings, we also analyzed the ERP data time-locked to the offset of the critical verb/action (see Holzgrefe-Lang et al., 2016, for a similar procedure). The pattern of results was largely identical to that of the analyses reported here, and those analyses can be found in the Supplementary Materials.

Processed data were exported to and analyzed in R (R Development Core Team, 2008) using the “ez” package (Lawrence, 2016). Separate repeated measures ANOVAs were run on the data from Experiment 1 and Experiment 2, with region (frontal vs. central vs. posterior) and condition (BC vs. NO) included as within-subject factors. The significance level was set to α = 0.05. In cases of violations of the sphericity assumption, Greenhouse-Geisser corrected values are reported. Furthermore, ERPs were plotted using the “ggplot2” package (Wickham, 2016), and plotted data were 8 Hz low-pass filtered for presentation purposes.

## Results

### Experiment 1: Prosodic Boundary Cues in Auditory Speech Sequences

#### Behavioral Data

In line with the analysis approach of Holzgrefe-Lang et al. (2016), we examined the button press responses to the bracketed boundary and no-boundary sequences that were presented after each auditory verb sequence using mixed-effects logistic regression models (using the lme4 package; Bates et al., 2015). We ran an intercept-only model for each condition, to examine whether participants’ correct response rate in each condition was above chance level (50%), including random intercepts for participant and item in both models. Participants offered no response on only 11 (out of a total of 2760) trials, and these trials were excluded from the models. On average, participants correctly identified the presence of a boundary on 96% (*SD* = 9%) of trials in the BC-condition, significantly above-chance performance, β = 4.94, *SE* = 0.65, *z* = 7.59, *p*< 0.001, and correctly identified the absence of a boundary on 99% (*SD* = 3%) of trials in the NO-condition, also significantly above chance, β = 6.68, *SE* = 1.17, *z* = 5.69, *p*< 0.001. These results confirm that the prosodic boundary cues were sufficient to signal the presence of a boundary in the BC-condition, and the absence of these cues was sufficient to signal that no boundary was present in the NO-condition.

#### ERP Data

**FIGURE 3:**
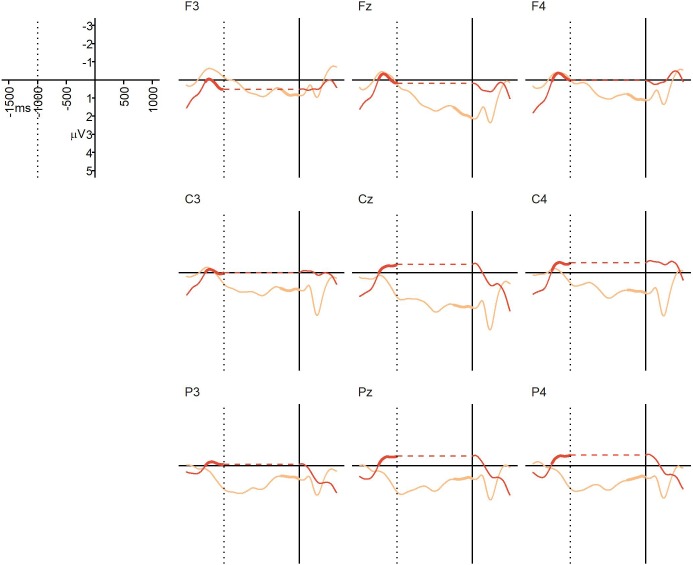
Experiment 1 (auditory speech sequences): grand average ERPs at representative electrodes in the NO-condition (no boundary, dark orange) and BC-condition (with boundary, light orange), time-locked to the onset of the post-boundary word “und” (solid vertical line). The dotted vertical line indicates the average offset of the critical verb in both conditions (i.e., the onset of the pause in the BC-condition). No pause was present in the NO-condition, so in order to align the ERPs from the two conditions to both the average offset of the critical verb and the onset of the final “und”, a gap indicating the duration of the pause has been inserted into the NO line, represented by the horizontal dotted line (this was done for visualization purposes only). Thickened lines indicate the time intervals used for comparison.

The average percentage of trials that were artifact-free and could be used for averaging was 96% in the NO-condition (*SD* = 4%, range = 81–100%), and 93% in the BC-condition (*SD* = 5%, range = 81–98%). Figure 3 displays the grand average ERPs from Experiment 1. At the descriptive level, a positivity generally emerged at or before the offset of the critical verb in the BC-condition, and continued until the onset of the post-boundary word “und”. This positivity was not visible in the NO-condition. The repeated measures ANOVA yielded a significant main effect of condition, *F*(1,22) = 6.06, *p* = 0.022, $\eta_{G}^{2}$ = 0.086, confirming that the mean ERP amplitude in the 250 ms prior to the onset of the post-final “und” was more positive in the BC-condition (*M* = 0.96 μV, *SD* = 2.33) than in the NO-condition (*M* = -0.24 μV, *SD* = 1.02). There was no significant main effect of region, *F*(2,44) = 1.91, *p* = 0.17, $\eta_{G}^{2}$ = 0.0087, and no significant interaction, *F*(2,44) = 0.90, *p* = 0.38, $\eta_{G}^{2}$ = 0.0031. Thus, we found the expected CPS-like positivity in response to the prosodic boundary cues in the BC-condition relative to the NO-condition. The absence of a region-by-condition interaction suggests that this positivity is broadly distributed, in line with previous CPS findings for auditory stimuli (e.g., Steinhauer et al., 1999; Bögels et al., 2011a; Holzgrefe et al., 2013; Holzgrefe-Lang et al., 2016).

### Experiment 2: Kinematic Boundary Cues in Visual Action Sequences

#### Behavioral Data

To examine the behavioral data, we ran an identical analysis to that of Experiment 1. Participants offered no response on only 15 (out of a total of 4416) trials, and these trials were excluded from the models. On average, participants correctly identified the presence of a boundary on 86% (*SD* = 12%) of trials in the BC-condition, significantly above-chance performance, β = 2.36, *SE* = 0.30, *z* = 7.89, *p*< 0.001, and correctly identified the absence of a boundary on 92% (*SD* = 9%) of trials in the NO-condition, also significantly above chance, β = 3.17, *SE* = 0.31, *z* = 10.38, *p*< 0.001. These results confirm that the kinematic boundary cues were sufficient to signal the presence of a boundary in the BC-condition, and the absence of these cues was sufficient to signal that no boundary was present in the NO-condition.

#### ERP Data

**FIGURE 4:**
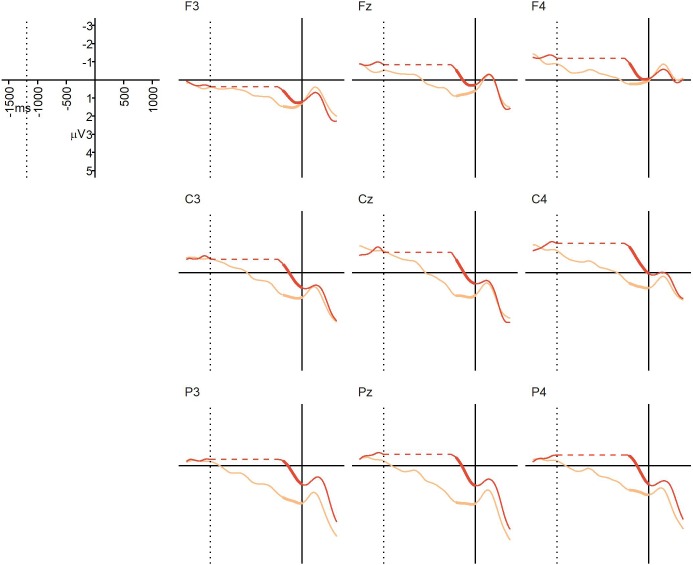
Experiment 2 (visual action sequences): grand average ERPs at representative electrodes in the NO-condition (no boundary, dark orange) and the BC-condition (with boundary, light orange), time-locked to the onset of the post-boundary action (solid vertical line). The dotted vertical line indicates the average offset of the critical action (i.e., the onset of the pause in the BC-condition). Unlike Experiment 1, a pause was present in both conditions, but the pause was significantly longer in the BC-condition than in the NO-condition. In order to align the ERPs from the two conditions to both the average offset of the critical action and the onset of the post-boundary action, a gap has been inserted into the NO-line, represented by the horizontal dotted line (this was done for visualization purposes only). Thickened lines indicate the time intervals used for comparison.

The average percentage of trials that were artifact-free and could be used for averaging was 94% in the NO-condition (*SD* = 11%, range = 55–100%), and 93% in the BC-condition (*SD* = 11%, range = 56–100%). Due to technical issues, data from the C4 electrode for one participant were corrupted, and data from this electrode for this participant were therefore removed from analyses and replaced by the mean value of the other electrodes from the same region and participant. Figure 4 displays the grand average ERPs from Experiment 2. At the descriptive level, a positivity emerged in the BC condition, and in some electrodes (e.g., Pz, Cz) this positivity began prior to the offset of the critical action. This positivity continued until the onset of the final action, which triggered an N1/P2 complex-like response. There was no such positivity in the NO-condition prior to the onset of the final action. The repeated measures ANOVA yielded a significant main effect of condition, *F*(1,22) = 12.02, *p* = 0.0022, $\eta_{G}^{2}$ = 0.10, confirming that the mean ERP amplitude during the 250 ms interval prior to the onset of the final action was more positive in the BC-condition (*M* = 1.20 μV, *SD* = 1.38) than in the NO-condition (*M* = 0.06 μV, *SD* = 1.51). There was no significant main effect of region, *F*(2,44) = 2.56, *p =* 0.11, $\eta_{G}^{2}$ = 0.023, and no significant interaction, *F*(2,44) = 2.21, *p* = 0.15, $\eta_{G}^{2}$ = 0.011. Thus, we found the expected positivity (i.e., a CPS-like ERP component) in response to the kinematic boundary cues in the BC-condition, but not in the NO-condition. The absence of a region-by-condition interaction suggests that this action-boundary CPS is broadly distributed. Overall, despite some differences in the mean amplitude prior to, and following the pause, the similarity between the ERPs in Experiment 1 and Experiment 2 is striking. Both show a clear positive shift in the BC condition that continues only until interruption by the onset of the final sub-unit. The results of the statistical analyses also suggest a similarity in timing of the positivity between the two conditions.

## General Discussion

The current study took an interdisciplinary approach by examining similarities in the bottom-up processing of perceptual boundary cues presented in speech and action. In two experiments, we presented two groups of adult participants either with auditory sequences of three spoken verbs co-ordinated with an “und”, or with visual sequences of three performed actions. In two within-subjects conditions, the sequences either did or did not contain prosodic (auditory speech sequences) or kinematic (visual action sequences) boundary cues to signal a boundary between the critical second verb or action and the following sub-unit. As expected, a prosodic boundary within the auditory verb sequences evoked a broadly distributed positivity in the ERP, which we interpret as a CPS (e.g., Steinhauer et al., 1999; Bögels et al., 2011a; Holzgrefe et al., 2013; Holzgrefe-Lang et al., 2016), confirming that the CPS is a robust marker of prosodic boundary processing in speech.

Critically, our study found a positivity that shared temporal and spatial characteristics with the CPS in response to kinematic boundary cues in visual action sequences: The positivity began prior to the onset of the post-boundary action, and was broadly distributed across frontal, central, and parietal electrodes. These characteristics are also in line with previous examinations that have found similar temporal and spatial distributions of the CPS to different speech stimuli (e.g., Steinhauer et al., 1999; Bögels et al., 2011a; Holzgrefe et al., 2013; Glushko et al., 2016; Holzgrefe-Lang et al., 2016). This finding thus suggests that the processes underlying prosodic boundary processing, and eliciting the CPS, are also operating during the processing of kinematic boundary cues. The similarity of the ERP in response to kinematic and prosodic boundary cues is further evidence that the CPS reflects bottom-up, domain-general perceptual chunking processes that underlie the segmentation of continuous input (Gilbert et al., 2015).

Previous work has shown a CPS-like positivity across different linguistic levels, domains, and modalities in which durational boundary cues are present: in response to boundaries within jabberwocky and hummed speech free from syntactical or lexical information (Pannekamp et al., 2005), in music (Glushko et al., 2016), and also to visually presented linguistic stimuli (silent reading of visually presented word-by-word sentences; e.g., Steinhauer, 2003; Hwang and Steinhauer, 2011). The current findings extend this work by showing a CPS-like positivity in response to perceptual boundary cues in visually presented non-speech stimuli. Notably, we chose a between-subjects design to prevent a “translation” or “transfer” of the auditory material in Experiment 1 to the visual material in Experiment 2. Hence, the current study further supports the domain-general explanation of prosodic boundary processing by demonstrating that the CPS is also evoked by kinematic boundary cues. Specifically, the current work suggests that the CPS is sensitive to the bottom-up processing of lower-level perceptual boundary cues present in observed action sequences. It is also important to note that the current study cannot rule out an effect of top-down processes, in part because we required participants to decide whether a boundary was present on each trial or not. However, the CPS has been previously found in response to prosodic speech boundaries in the absence of such a task (e.g., Holzgrefe et al., 2013), which could indicate that the CPS is also elicited in natural passive listening paradigms. However, future research is necessary to verify whether a CPS is also elicited by passive viewing of actions sequences without an explicit task. Previous neuroimaging work has also suggested that the CPS reflects the attentional and memory processes involved in segmentation (Knösche et al., 2005), which in light of the current study suggests that these domain-general processes support the segmentation of incoming information, independent of the domain being auditory or visual.

There has been some difficulty with identification of the CPS in previous work, and some researchers have claimed that previously reported examples of the CPS are in fact due to so-called “obligatory onset components” related to processing of the post-boundary sub-unit (e.g., Männel and Friederici, 2009). By time-locking the ERP to the onset of the post-boundary word/action and running our analyses in the time interval prior to this onset, we can be sure that the positivity was in response to the boundary, and was uncontaminated by any response to the final sub-unit (i.e., the third element; see Supplementary Materials for an analysis time-locked to the offset of the critical verb/action with a largely identical pattern of results). This approach was particularly important given that the current study presented participants with naturally produced stimuli, meaning that the time interval between the offset of the critical sub-unit and the onset of the final sub-unit varied substantially across trials. Use of naturally produced stimuli also meant that any boundary cues within the sequences were not controlled or manipulated, but analyses showed that the duration of the critical action and the pause between the critical and final action were lengthened in the BC-condition relative to the NO-condition. While in the speech domain, it has been shown that the pause is not relevant for the elicitation of a CPS (Steinhauer et al., 1999; Holzgrefe-Lang et al., 2016) and that a combination of lengthening and pitch-rise, but not the single boundary cues in isolation, is necessary to elicit a CPS (Holzgrefe-Lang et al., 2016), the effect of these cue combinations on processing of a kinematic boundary is still unknown. A vital next step is therefore to pursue a fine-grained analysis of the kinematic boundary cues that are necessary and sufficient to reliably signal a boundary in the action domain.

The shared features of the kinematic and the prosodic boundary cues in the current study were durational: both extended the duration of the critical sub-unit, and extended the time interval between the critical and final sub-units. This work thus suggests that these temporal cues are sufficient to drive the perceptual chunking processes underlying the segmentation of both speech and action. This suggestion is supported by recent work showing that durational cues are sufficient to support segmentation of abstract streams of information, such as sequences of patterns. For example, Frost et al. (2017) presented adult participants with a stream of visual patterns and found that lengthening the duration of single patterns encouraged participants to perceive a boundary at that position and form a segment from the previously presented patterns. It is therefore conceivable that the CPS reflects bottom-up processing, which can be driven by these lower-level perceptual cues alone. A key challenge for future research is thus to determine the extent to which the bottom-up processing of perceptual boundary cues interacts with top-down constraints imposed by the listener/observer (e.g., contextual information; prior knowledge; tracking of the actor’s intention) to determine appropriate segmentation of the input stream. Furthermore, that our results indicate domain-general segmentation processes fits well to the fact that sign language conveys certain communicative (or linguistic, e.g., prosodic) content by means of kinematic cues (Malaia et al., 2013), which require cross-domain integration of incoming information.

Overall, the present study shows that the electrophysiological correlates of speech segmentation are similar to those involved in the segmentation of action sequences, which suggests that the processing of prosodic and kinematic boundary cues might rely on similar underlying processes. Given the similarities between speech and action, both are continuous information streams organized hierarchically that convey similar perceptual boundary cues, it would stand to reason that the processes supporting the processing of these streams are shared. Accordingly, this interdisciplinary research approach provides yet further evidence that the processing of prosodic boundary cues in speech and kinematic boundary cues in action for the purpose of segmentation is driven by domain-general mechanisms.

## Data Availability

The datasets generated for this study can be found on the Open Science Framework, doi: 10.17605/OSF.IO/PQ8XZ.

## Ethics Statement

This study was carried out in accordance with the recommendations of the ethics committee of the University of Potsdam with written informed consent from all participants. All participants gave written informed consent in accordance with the Declaration of Helsinki. The protocol was approved by the ethics committee of the University of Potsdam (approval number 16/2015).

## Author Contributions

MH and RR wrote the manuscript. All authors were involved in study design, data collection, analyses, and contributed intellectually to the manuscript.

## Conflict of Interest Statement

The authors declare that the research was conducted in the absence of any commercial or financial relationships that could be construed as a potential conflict of interest.

## Footnotes

1 To explore whether the CPS can be elicited by a mere inclusion of a silent pause, each participant also listened to two further conditions, which were created from the NO-condition and included artificial pauses at unnatural positions. An artificial pause with a duration of 1000 ms (based on the mean pause duration in the BC-condition) was inserted either between the second (i.e., pre-boundary) verb and the post-boundary conjunction (60 sequences), or between the post-boundary conjunction and the third (i.e., post-boundary) verb (60 sequences). The ERPs in these unnatural conditions showed no CPS. Participants’ average boundary detection on unnatural boundary trials was unrelated to their boundary detection on natural boundary trials, *r*_s_ (21) = 0.16, *p* = 0.45. Since these conditions are not under the scope of the present article as they are not comparable to the action sequences, we exclusively present details of the stimuli and corresponding results of the 120 verb sequences of the natural NO- and BC-conditions.

2 http://www.neurobs.com

3 http://www.cedrus.com

4 This baseline interval was chosen as the latest sensible time interval in which the stimuli of both conditions were identical. This baseline interval, however, meant that the baseline and analysis time intervals partially overlapped in Experiment 1. See Supplementary Materials for an analysis in which this partial overlap was removed, yielding comparable results.
