# Supplementary material for: Parallels in Processing Boundary Cues in Speech and Action
Source: Front Psychol. 2019 Jul 16;10:1566. doi: 10.3389/fpsyg.2019.01566 (PMC6646704; doi:10.3389/fpsyg.2019.01566)
Supplement: Supplementary file 1 [file Table_1.docx]

Experiment 1 (auditory speech sequences):

A repeated measure analysis of variance (ANOVA) was run to examine whether mean ERP amplitude differed by condition (NO vs. BC) and region (frontal vs central vs posterior) during the 250 ms following the offset of the critical verb. This yielded a significant main effect of condition, *F*(1,22) = 6.42, *p* = .019, $\eta_{G}^{2}$ = 0.065, confirming that the mean amplitude was more positive during the 250 ms interval following the offset of the critical action in the BC-condition (*M* = 0.94 µV, *SD* = 1.76) than in the NO-condition (*M* = 0.0066 µV, *SD* = 1.35). There was no significant main effect of region, *F*(2,44) = 1.39, *p* = .25, $\eta_{G}^{2}$ = 0.011, but a significant interaction was found, *F*(2,44) = 19.87, *p* < .001, $\eta_{G}^{2}$ = 0.057. Follow-up contrasts revealed that mean amplitude was significantly more positive in the BC than NO-condition among posterior electrodes (*p* < .01) and central electrodes ( *p* <.01), but not among frontal electrodes ( *p =* .99). Thus, in the time window immediately following the offset of the critical verb, a positivity in the BC-condition was found mainly over central and posterior electrodes. Similar to our findings from the initial “backward” analysis, we found the expected positivity (i.e., a CPS-like ERP component) in response to the prosodic boundary cues in the BC-condition, but not in the NO-condition.


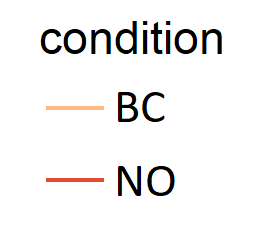


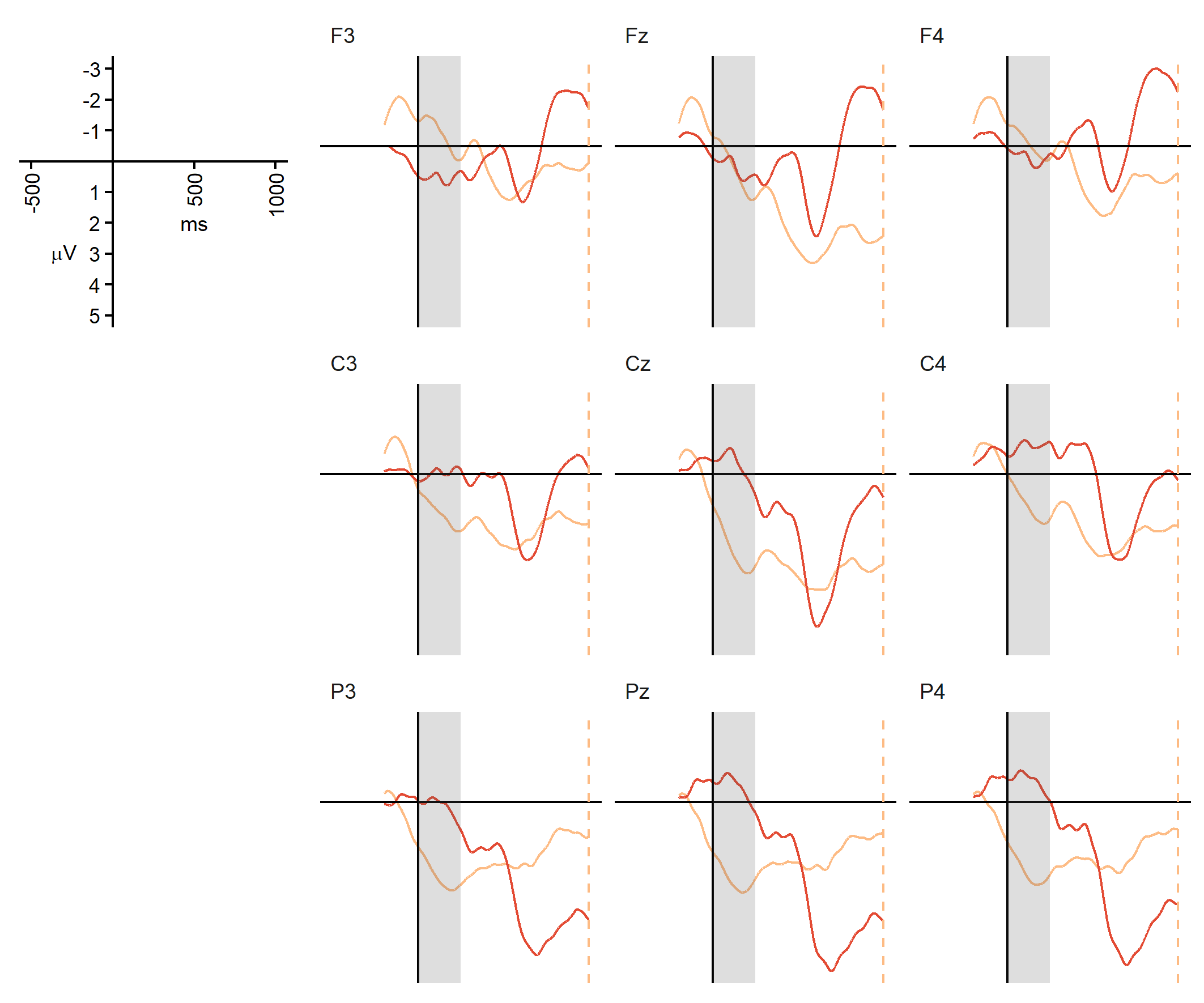


Figure 1. Experiment 1 (auditory speech sequences): Grand average ERPs at representative electrodes in the NO-condition (no boundary, dark orange) and BC-condition (with boundary, light orange), time-locked to the offset of the critical word (y-axis). The dotted vertical line indicates the onset of the final “und” (and) in the BC-condition. The 250 ms interval during which analyses were run is shaded grey.

Experiment 2 (Action sequences):

Identical analyses were also run on the mean ERP amplitude in the 250 time interval following the offset of the critical action. The repeated measures ANOVA yielded a significant main effect of condition, *F*(1,22) = 5.50, *p* = .028, $\eta_{G}^{2}$= .035, confirming that the mean amplitude was more positive during the 250 ms interval following the offset of the critical action in the BC-condition (*M* = -0.61 µV, *SD* = 1.21) than in the NO-condition (*M* = -1.12 µV, *SD* = 1.12). There was also a main effect of region, *F*(2,44) = 9.53, *p =* .002, $\eta_{G}^{2}$ = 0.068, with follow-up contrasts indicating that mean amplitude was significantly more positive in frontal electrodes than in central electrodes (*p* < .01) and in posterior electrodes than central electrodes (*p* < .001), while there was no statistically significant difference in mean amplitude between anterior and posterior electrodes (*p* > .99). No significant interaction between region and condition was found, *F*(2,44) = 0.0072, *p* = .95, $\eta_{G}^{2}$< 0.001. Thus, similar to our findings from the initial “backward” analysis, we found the expected positivity (i.e., a CPS-like ERP component) in response to the kinematic boundary cues in the BC-condition, but not in the NO-condition.
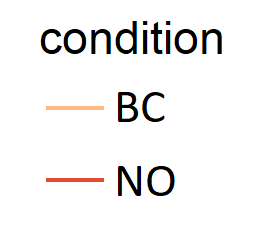

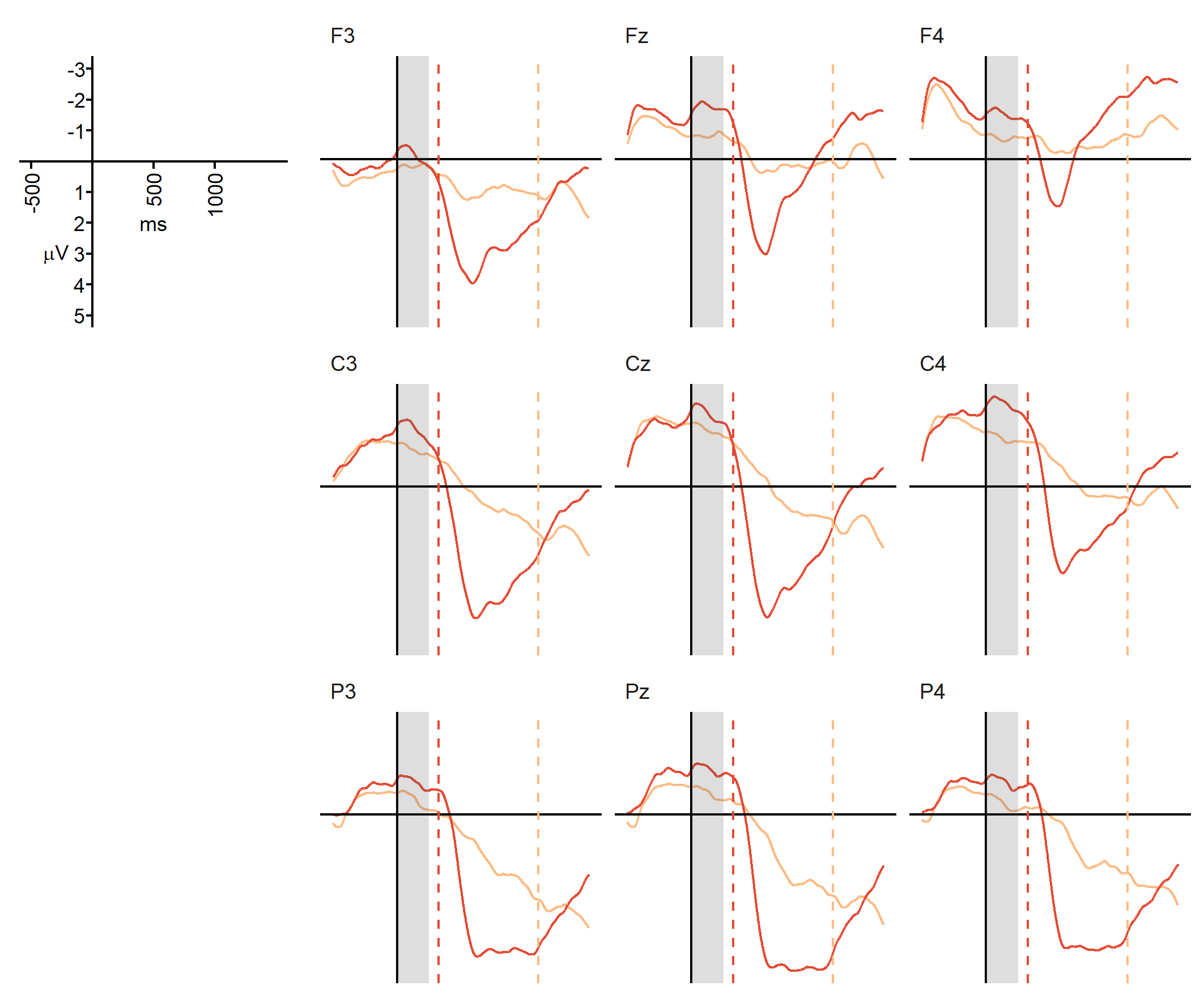


Figure 2. Experiment 2 (action sequences): Grand average ERPs at representative electrodes in the NO-condition (no boundary, dark orange) and BC-condition (with boundary, light orange), time-locked to the offset of the critical word (y-axis). The dotted vertical lines indicate the average onset of the final action in both the BC- and NO-condition color-coded according to the legend. The 250 ms interval during which analyses were run is shaded grey.
